# Genetic Testing among Children in a Complex Care Program

**DOI:** 10.3390/children4050042

**Published:** 2017-05-22

**Authors:** Krista Oei, Robin Z. Hayeems, Wendy J. Ungar, Ronald D. Cohn, Eyal Cohen

**Affiliations:** 1 Faculty of Medicine, University of Toronto, Toronto, ON M5S 1A8, Canada; krista.oei@mail.utoronto.ca; 2 Division of Paediatric Medicine, Hospital for Sick Children, Toronto, ON M5G 1X8, Canada; 3 Child Health Evaluative Studies, Hospital for Sick Children, Toronto, ON M5G 1X8, Canada; robin.hayeems@sickkids.ca (R.Z.H.); wendy.ungar@sickkids.ca (W.J.U.); 4 Institute of Health Policy and Management and Evaluation, University of Toronto, Toronto, ON M5T 3M6, Canada; 5 Department of Paediatrics, University of Toronto, Toronto, ON M5G 1X8, Canada; ronald.cohn@sickkids.ca; 6 Program in Genetics and Genomic Biology, Hospital for Sick Children, Toronto, ON M5G 1X8, Canada

**Keywords:** children with medical complexity, complex care, genetic testing, health care utilization

## Abstract

Little is known about the pattern of genetic testing and frequency of genetic diagnoses among children enrolled in structured complex care programs (CCPs). Such information may inform the suitability of emerging genome diagnostics for this population. The objectives were to describe the proportion of children with undiagnosed genetic conditions despite genetic testing and measure the testing period, types and costs of genetic tests used. A retrospective analysis of 420 children enrolled in Toronto’s Hospital for Sick Children’s CCP from January 2010 until June 2014 was conducted. Among those who underwent genetic testing (*n* = 319; 76%), a random sample of 20% (*n* = 63) was further analyzed. A genetic diagnosis was confirmed in 48% of those who underwent testing. Those with no genetic diagnosis underwent significantly more genetic tests than those with a confirmed genetic diagnosis [median interquartile range (IQR): six tests (4–9) vs. three tests (2–4), *p* = 0.002], more sequence-level tests and a longer, more expensive testing period than those with a genetic diagnosis [median (IQR): length of testing period: 4.12 years (1.73–8.42) vs. 0.35 years (0.12–3.04), *p* < 0.001; genetic testing costs C$8496 ($4399–$12,480) vs. C$2614 ($1605–$4080), *p* < 0.001]. A genetic diagnosis was not established for 52% of children. Integrating genome-wide sequencing into clinical care may improve diagnostic efficiency and yield in this population.

## 1. Introduction

Children with medical complexity (CMC) have chronic conditions resulting in high family-identified service needs, functional impairments, multiple subspecialist involvement, and elevated health service use [1]. These children comprise <1% of the pediatric population, but account for about one-third of pediatric health care spending [2]. In response to the intensity and expense of their care as well as parental reports of unmet need, structured complex care programs (CCP) have rapidly emerged to coordinate and improve their care [1,3,4]. Although the types of children enrolled in CCPs vary, virtually all focus on children with multiple morbidities. Complex neurologic phenotypes and/or a suspected or confirmed genetic condition are common in many [5]. Genetic testing is often pursued to establish a unifying diagnosis, understand pathogenicity and disease progression, guide care, and inform reproductive planning. However, a definitive molecular diagnosis is not always found in CMC, despite efforts to confirm clinical suspicions of an underlying genetic condition. For many families, these diagnostic odysseys are time, resource, and emotionally intensive [6,7,8,9,10].

As the number of clinically available genome-based tests grows, optimizing their application for CMC warrants consideration. Improved detection rates [11,12,13] and decreasing laboratory costs [14] are driving the adoption of exome and genome sequencing (i.e., technology that can analyze an individual’s whole exome/genome in a single test) in clinical care around the world [15,16,17]. While early studies in some cohorts (e.g., autism spectrum disorders [11]) are promising, evidence to inform potential utility in broad groups of CMC has not been generated. 

The objective of this study was to describe the use of genetic testing in a cohort of children in a structured CCP. Specifically, in order to gauge the potential suitability of exome/genome sequencing-based technologies for routine use in CCPs, we aimed to determine the proportion of children remaining without a confirmed diagnosis despite undergoing genetic testing, as well as to characterize the duration of the genetic testing journey, and the types and costs of genetic tests used.

## 2. Methods 

### 2.1. Setting

The CCP at the Hospital for Sick Children (SickKids) was established in 2006 and is the largest structured CCP in Canada; over 550 children at SickKids and five community-hospital locations have been enrolled since inception. Clinical care is provided by an interprofessional complex care team comprised of a nurse practitioner, a complex care physician, and other allied healthcare professionals [18,19]. Children with medical complexity are eligible for enrolment in the CCP provided they meet all of the following criteria: (1) are below 16 years of age, (2) require care coordination, (3) have a primary care provider that will remain actively involved in their care, (4) are actively followed by a SickKids subspecialist, (5) are not already part of a comprehensive multi-disciplinary program (e.g., cystic fibrosis). They must also have a severe underlying chronic health condition, have a life expectancy of at least 6 months, require the involvement of multiple healthcare practitioners and be technology dependent and/or users of high intensity care. Inclusion criteria of this CCP which emphasize the complexity, chronicity and fragility of this patient population are similar to other complex care clinical programs in North America [5]. Genetic testing is ordered, where indicated, by a consulting geneticist and occasionally by other subspecialists.

This study protocol was approved by the Research Ethics Board at the Hospital for Sick Children, and a waiver of consent was obtained for this retrospective review.

### 2.2. Study Cohort 

Children were identified for inclusion from SickKids’ Complex Care Database, based upon CCP enrolment from the date of database inception (1 January 2006) until 11 June 2014. Children discharged from the CCP or deceased prior to 2010 were excluded in an effort to ensure that all participants had had the opportunity to pursue chromosome microarray. In 2010, chromosome microarray analysis became the recommended first-line test for the diagnostic assessment of children with multiple anomalies, non-syndromic developmental and intellectual disabilities and autism spectrum disorders [20], and was adopted locally. As such, children who only underwent genetic testing prior to 2010 were excluded to provide an accurate reflection of post-microarray genetic testing practice. The younger siblings of families with multiple siblings enrolled in the CCP were excluded as their genetic testing may have been targeted according to their older sibling’s test results, underestimating an individual’s typical test use. The remaining 420 children were then divided into those who underwent genetic testing (*n* = 319) and those who did not (*n* = 101). Using computer-generated random numbers, 20% of the children who underwent genetic testing were selected to form the study cohort of 63 children and were further divided into two groups: those for whom a genetic diagnosis was confirmed and those with no genetic diagnosis. A confirmed genetic diagnosis was defined as a diagnosis validated by a positive genetic test result that explained all major clinical findings. For most cases, genetic diagnoses were confirmed in the genetic consultation letters of the patient charts and all cases were then subsequently reviewed by a clinical geneticist to verify variant causation. All other children who underwent genetic testing but did not meet these criteria were classified as no genetic diagnosis. Included among those classified as no genetic diagnosis were children for whom variants of unclear significance were found on genetic testing but who did not have a positive genetic test result explaining major clinical findings. To gain a better understanding of the overall genetic diagnoses of the children in the entire CCP and to determine the total number of children in the CCP that remained without a diagnosis despite undergoing genetic testing, the diagnoses of the entire population of children that underwent genetic testing (the parent sample) were also further described. Similar to the study cohort, the children in the parent sample were divided into confirmed genetic diagnosis and no genetic diagnosis. The diagnoses of those that received a confirmed genetic diagnosis were then divided into groupings based on the type of genetic diagnosis.

### 2.3. Data Collection Procedures and Measures

#### 2.3.1. Data Sources

Patient-level data were obtained from care plan summaries for each child, housed in the Hospital’s Complex Care Database and Electronic Patient Charts. 

#### 2.3.2. Demographic and Clinical Characteristics

We collected information on date of birth, sex, ethnicity, location of care, prematurity status, birth weight, primary diagnosis, congenital anomalies, developmental delay, technology assistance, genetic tests pursued and genetic test results. Congenital anomalies were defined using the European Registry of Congenital Anomalies and Twins (EUROCAT), Version 2014 [21]. The number of organ systems with at least one congenital anomaly was categorized for each child based on EUROCAT, and subdivided into single organ congenital anomalies and those affecting multiple organs. Detailed chart review captured the total number and types of genetic tests ordered for each child, name and location of testing laboratory (for purpose of ascertaining test prices), test results, genetic diagnosis, date of first consultation that included the ordering of a genetic test, and date of laboratory test results. 

#### 2.3.3. Genetic Tests

Genetic test types included all cytogenetic and molecular genetic tests that were conducted for diagnostic purposes and those conducted as part of a research study. There were no duplications between genetic tests performed as part of a research study and those that were available clinically. Research tests were included since newer generations of genetic tests are often available on a research basis prior to their clinical availability and can be integral to the diagnostic quest. Biochemical tests that were conducted either for diagnosis (e.g., metabolic panels) or post-diagnosis (i.e., for disease monitoring) were excluded. Each genetic test ordered constituted a single test, even if multiple genes were included in a gene panel test. Genetic tests were first characterized as one of the following nine types: (1) karyotype, (2) genome/exome-wide microarray, (3) targeted dosage tests (i.e., fluorescence in situ hybridization, multiplex ligation-dependent probe amplification), (4) Sanger sequencing for single gene and gene panel tests, (5) next-generation sequencing-based gene panel tests (6) recurrent mutation tests, (7) exome sequencing, (8) chromosome instability tests and (9) other (e.g., triplet repeat, mitochondrial disease DNA-based tests). Tests were then categorized into one of three mutually exclusive groupings: copy number variant and dosage tests (1, 2, 3), sequence-level tests (4, 5, 6, 7) or other types of tests (8, 9). The length of the testing period was defined as the period between the date of the first clinical genetics consultation and the laboratory report date for the positive genetic test result. Where the laboratory report was not available, the date the genetic diagnosis was communicated to the patient and/or family was used. For children for whom a genetic diagnosis had not yet been established, duration of testing data was censored at the end date of 30 June 2014. If the child was deceased or was lost to follow-up before a diagnosis was determined, the testing period was determined to have ended at that time (i.e., date of death and date of last attended appointment, respectively).

### 2.4. Costs

Costs for genetic testing per child were determined from the perspective of the payer (provincially-administered universal health care system, Ontario’s Ministry of Health and Long-Term Care). Test costs were represented by the charge or fee for genetic tests conducted at SickKids, in laboratories elsewhere in Canada, or in US/Europe. Charges were summed to determine total costs per child up until 30 June 2014. As clinical (but not research) genetic testing is reimbursed by the public provincial health insurance plan, the charges for genetic tests conducted at US/European labs were based upon the amount charged to the payer at the time the test was conducted. Foreign currencies were converted using the average foreign exchange rate of the transaction year [22]. In Canada’s provincially-administered universal health care system, routine genetic tests are covered by the provincial public insurance providers. In Ontario, for clinical genetic tests that are warranted but are not licensed in Canada (e.g., whole exome sequencing), exceptional access to these tests can be granted on a case by case basis. In this study, the costs associated with these out-of-country tests were individually reviewed, approved and covered by the Ontario Ministry of Health and Long-Term Care. Test charges were converted to 2015 Canadian dollars using the Consumer Price Index for health goods [23]. For genetic tests conducted at SickKids, the charges established by the Genome Diagnostics Laboratory were used. As such, all test charges included test costs, lab interpretation costs, and an institutional mark-up. Actual dollars paid by the payer were used where these figures were available. Current charges were used in all other situations. Any associated parental testing, genetic counseling or physician consultation fees were excluded. Where genetic test charges at a specific lab could not be obtained, a charge for the equivalent test conducted at a comparable testing facility was used. The charges of six rare single gene sequencing tests could not be obtained and as such an average of all single gene sequencing charges in the sample was used to approximate the charge of these six tests. Test prices ranged from C$202 to C$4720 for copy number variant and dosage tests, C$157 to C$8475 for sequence-level tests, and C$304 to C$8894 for other types (all reflective of 2015 Canadian dollars). 

### 2.5. Analysis

The primary analysis was conducted on the random sample of 63 cases for whom detailed chart abstraction and cost calculations were performed. Categorical data were compared between the children for whom a genetic diagnosis was and was not confirmed using Chi-Squared tests. The number of genetic tests performed, length of testing period, and the dollars spent on genetic testing were compared between the children for whom a genetic diagnosis was and was not confirmed using Mann–Whitney U tests as the data were non-normal. All analyses were conducted using SPSS Version 20.0 (Armonk, NY, USA). Values with *p* < 0.05 were considered to be significant.

## 3. Results

**Figure 1:**
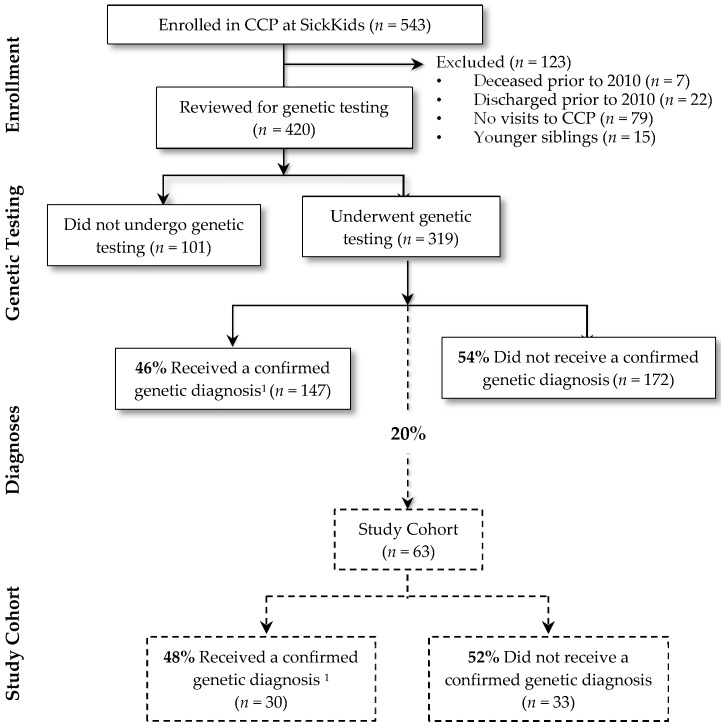
Study cohort derivation. ^1^ Subjects obtained a positive genetic test result that was related to clinical findings. CCP, complex care program.

Figure 1 summarizes the study cohort derivation. There were 543 children who were enrolled in the CCP at SickKids, of which 108 were inactive. Fifteen were younger siblings and thus excluded from the study. Of the remaining 420 children, 319 (76%) children underwent genetic testing. Of these, a genetic diagnosis was confirmed in 147 (46%). Genetic diagnoses were heterogeneous; the most common diagnoses included Trisomy 21 (*n* = 18; 12%), 22q11 deletion syndrome (*n* = 6; 4%) and CHD7 confirmed CHARGE syndrome (*n* = 5; 4%). Of the 63 cases for whom a detailed chart review was conducted, a genetic diagnosis was confirmed for close to half (*n* = 30; 48%). The remaining 33 (52%) had no genetic diagnosis established. This proportion is similar to the entire group of 319 children that underwent genetic testing in the entire complex care program; of these children, 172 (54%) had no genetic diagnosis established.

### 3.1. Characteristics of the Cohort (n = 63)

Demographic and clinical characteristics are summarized in Table 1. The largest proportion of children in the study cohort were 0–4 years of age (47%) and 54% were male. Multiple congenital anomalies were common, of which the most frequent were congenital heart defects and nervous system congenital anomalies, and most had functional limitations requiring assistance from technology (78%). No large differences in baseline demographics (age, sex, ethnicity, and location) and clinical characteristics (prematurity, birth weight, developmental delay, congenital anomalies, technology assistance, and start date of testing period) were noted between the children for whom a genetic diagnosis was established compared with those for whom it was not. Furthermore, baseline demographics and proportion of children who received a confirmed genetic diagnosis in the study cohort were similar to the source population (all 319 children who underwent genetic testing in the CCP).

### 3.2. Genetic Tests

The quantity, length of testing period and costs for genetic testing are summarized in Table 2. The number of genetic tests performed on children with no genetic diagnosis was significantly greater than those children with a confirmed genetic diagnosis (median interquartile range (IQR): six tests (4–9) vs. three tests (2–4) respectively, *p* = 0.002).

### 3.3. Length of Testing Period

The length of the genetic testing period was longer for those children for whom a genetic diagnosis was not confirmed [median (IQR): 4.12 years (1.73–8.42)] compared to those for whom a genetic diagnosis was confirmed [median (IQR): 0.35 years (0.12–3.04)], *p* < 0.001 (Table 2). 

Both groups had a median start date (first clinical genetics consultation) of September 2009. 

### 3.4. Genetic Testing Costs

Costs for genetic testing per child were higher among children with no genetic diagnosis [median (IQR): C$8496 (C$4399–C$12,480)] compared to those children for whom a genetic diagnosis was confirmed [median (IQR): C$2614 (C$1605–C$4080)], *p* < 0.001 (Table 2). Sequence-level tests (category types 4, 5, 6, 7, outlined below in Methods) of specific genes were the most expensive type of test (ranging from C$157 to C$8475) and were largely conducted in international laboratories, substantially increasing resources spent for children for whom a genetic diagnosis could not be confirmed.

### 3.5. Types of Genetic Testing

**Figure 2:**
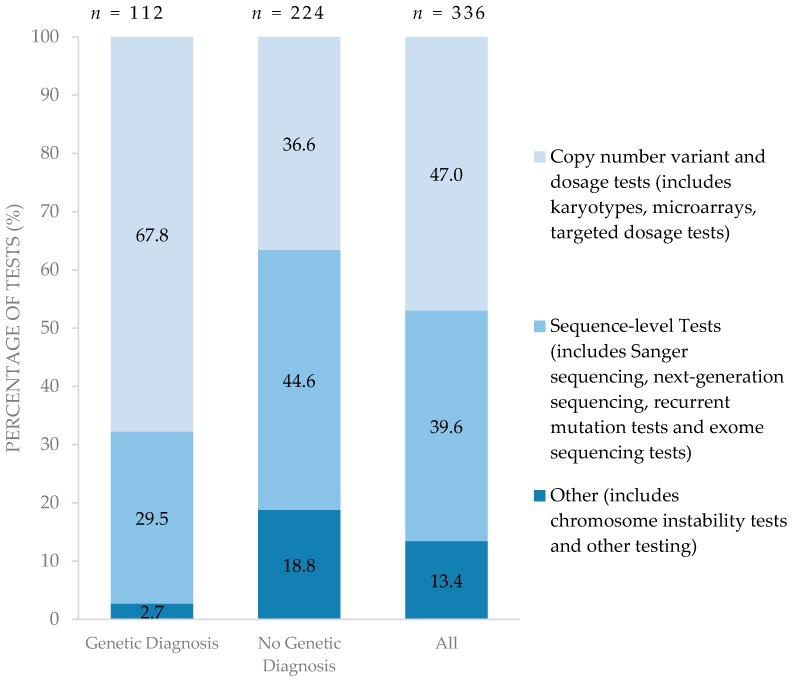
Type of testing performed among those with and without confirmed genetic diagnoses. (*n* = number of tests).

Figure 2 summarizes the types of genetic testing performed for each diagnostic group. Compared to children for whom a diagnosis was established, children with no genetic diagnosis pursued a greater proportion of higher resolution testing such as sequence-level testing in search of a diagnosis. Of the 224 genetic tests performed on the 33 children with no genetic diagnosis, 44.6% were sequence-level tests. On the other hand, lower resolution tests such as copy number variant and dosage tests were able to establish a genetic diagnosis among those for whom genetic diagnoses were confirmed. Of 112 genetic tests performed on 30 children with a confirmed genetic diagnosis, 67.8% were conventional copy number variant/dosage sensitive tests. Only one child in the entire cohort underwent whole exome sequencing.

## 4. Discussion

This study describes the high utilization of genetic testing among children enrolled in a large structured CCP. The majority of children used high volumes of genetic testing (median of four genetic tests) over a median time period of greater than 2 years and most (52%) remained undiagnosed. Compared to those for whom a diagnosis was achieved, undiagnosed children pursued a greater number of genetic tests overall and a greater number of high-resolution tests such as sequence-level testing.

Our findings suggest that genetic testing is utilized at high rates in complex pediatric populations. Other studies have characterized the use of all diagnostic tests (i.e., not restricted to genetic tests) in neurology clinics for children with both broad [27] and specific [28] neurological phenotypes. Similarly, they report high costs of diagnostic investigations (C$4428–C$16,349), modest diagnostic yield (6–31%) and a long duration of testing (mean of 40 months). Our findings focus specifically on patients enrolled in a CCP, many of whom have neurologic phenotypes. However, in our cohort, the majority also had congenital anomalies and more complex health needs than those described in these other populations. Given the increasing emphasis on optimizing clinical delivery pathways for the CMC, our findings provide a benchmark to assess the suitability of exome/genome sequencing technologies for this population.

The suitability of exome/genome sequencing incorporates a combination of test performance, cost, and acceptability to the population. A recent costing study of next-generation sequencing for autism spectrum disorder estimated costs to be C$1655 (95% CI: C$1611, C$1699) for whole exome sequencing and C$2851 (95% CI: C$2750, C$2956) or C$5519 (95% CI: C$5244, C$5785) [29], depending upon the sequencing platform used, for whole genome sequencing. These single test costs (in a less complex population) fall below the median total dollars spent on testing for the undiagnosed group reported herein. However, test performance and acceptability within this population warrant consideration before exome/genome sequencing may be deemed the favourable testing option. 

A number of study limitations are notable. This was a single center study. Although this program shares similarities in patient characteristics with other complex programs (e.g., complexity, fragility and functional limitations), findings may differ in other CCPs based on their specific population. Furthermore, costing estimates and coverage of genetic testing reflected those of the province of Ontario, where whole exome sequencing and whole genome sequencing are not yet licensed as clinical tests, and may differ in other jurisdictions. The primary analysis was conducted on a random sample; although the characteristics of the sample were similar to those of the total clinic, nevertheless, this may have led to some degree of imprecision of estimates. Duration of follow-up and types of genetic tests available to the children enrolled in the CCP were not consistent over the study period. However, children with no genetic diagnosis and children with a confirmed genetic diagnosis had similar median start dates for testing (September 2009) so this lead time bias did not affect the length of follow-up of the two groups. Six cases in the undiagnosed group died or were lost to follow-up and may have been a source of attrition bias. If anything, this shortened follow-up interval in the undiagnosed group would lead to an underestimation in duration of testing for this group. Additionally, charges including mark-ups were used to approximate dollars spent on all genetic testing (i.e., both international and provincial) and may over-estimate actual costs. We were unable to consistently ascertain whether charges reflected actual expenditures from the payer’s perspective. Although this study demonstrated that substantial charges for genetic testing are incurred for children enrolled in CCPs, the total costs may reflect a conservative estimate as several associated costs were omitted: metabolic testing, physician consultation and allied health service use (e.g., genetic counseling), parental testing, targeted testing and counseling for other family members, and costs borne by parents (e.g., travel and work loss). Furthermore, for patients with no diagnosis, the total number and costs of genetic tests excluded future tests that could be conducted after the study end date as they continued on their diagnostic quest.

Despite these limitations, this study highlights the resource intensity and time associated with conventional genetic testing in a CCP. Currently, chromosome microarray remains the recommended first tier diagnostic tool for children with multiple congenital anomalies and developmental delay [20]. As such, the diagnostic quest for children in this cohort generally began with a microarray or a hypothesis-driven targeted test where a specific syndrome is suspected. Typically, when preliminary tests do not confirm a diagnosis, the diagnostic quest proceeds in a step-wise manner. Specific genes are sequenced based on a revised diagnostic hypothesis, waiting for the test results, and then repeating the process with a further revised hypothesis. This can result in lengthy and expensive journeys [27,28,29]. As reported herein, as whole exome and whole genome sequencing are not yet licensed and/or widely used as clinical tests in some jurisdictions, these lengthy resource-intensive journeys continue to persist and support the need to adopt higher-resolution, hypothesis-free approaches to genetic diagnoses for CMC. Combined with the emotional weight known to be experienced by families awaiting diagnoses [6,7,8,9,10], this study highlights the need to improve the efficiencies of genetic testing and the overall experience of searching for a diagnosis for the growing number of children and families enrolled in CCPs.

## Figures and Tables

**Table 1:** Demographics and Clinical Characteristics of Cohort ^1^.

			Diagnosis				
	All Subjects		Confirmed Genetic Diagnosis		No Genetic Diagnosis		*p* Value ^2^
	*n* = 63		*n* = 30		*n* = 33		
**Demographics ^3^:**							0.97
Age group, *n* (%)							
0–4 years	30	(47)	14	(47)	16	(49)	
5–9 years	20	(32)	10	(33)	10	(30)	
10–18 years	13	(21)	6	(20)	7	(21)	
Sex, *n* (%)							0.27
Male	34	(54)	14	(47)	20	(61)	
Female	29	(46)	16	(53)	13	(39)	
Ethnicity, *n* (%) ^4^							0.03
Caucasian	17	(27)	5	(17)	12	(36)	
Non-Caucasian	30	(48)	19	(63)	11	(33)	
Location							0.59
Toronto ^5^	42	(67)	21	(70)	21	(64)	
Other	21	(33)	9	(30)	12	(36)	
**Clinical Characteristics:**							
Prematurity ^6^							0.30
Premature (<37 weeks) ^1^	12	(19)	<5		8	(24)	
Term (≥37 weeks)	50	(79)	25	(83)	25	(76)	
Birth weight ^7^							0.89
<2.5 kg	15	(24)	7	(23)	8	(24)	
≥2.5 kg	43	(68)	21	(70)	22	(67)	
Developmental Delay ^8^							0.72
Yes	58	(92)	28	(93)	30	(91)	
No ^1^	5	(8)	<5		<5		
No. organ systems involved in congenital anomalies ^9^							0.62
<2	40	(64)	20	(67)	20	(61)	
≥2	23	(36)	10	(33)	13	(39)	
Technology Assistance ^10^							0.69
Yes	49	(78)	24	(80)	25	(76)	
No	14	(22)	6	(20)	8	(24)	
Start Date of Testing Period ^11^:							0.48
Prior to 1 January 2010	37	(59)	19	(63)	18	(55)	
1 January 2010–30 June 2014	26	(41)	11	(37)	15	(45)	
Genetic Diagnoses in Total CCP ^12^							
Trisomy 21			18	(12)			
DiGeorge syndrome			6	(4)			
CHARGE syndrome ^13^			5	(4)			
Other			118	(80)			

^1^ All values are reflected as *n* (%) unless otherwise noted. To preserve confidentiality, cell sizes were only reported where *n* ≥ 5. ^2^ *p*-Values represent the statistical baseline differences between the confirmed genetic diagnosis and those children with no genetic diagnosis. ^3^ Baseline demographics of the total sample (all 319 children who underwent genetic testing in the CCP): sex (Male: 53%, Female: 47%), age (0–4 y: 40%, 5–9 y: 34%, 10–18 y: 26%), location (Toronto ^5^: 53%, Other: 47%); and diagnosis (confirmed genetic diagnosis: 46%, no genetic diagnosis: 54%). ^4^ Unknown for *n* = 16 (25%). ^5^ Toronto refers to the census metropolitan area of Toronto, Ontario, according to Statistics Canada. This area encompasses the City of Toronto and surrounding subdivisions totaling a land area of 5906 square km [24] and a population of 6.123 [25] million people in 2015. ^6^ Unknown for *n* = 1 (2%). ^7^ Unknown for *n* = 5 (8%). ^8^ Defined according to Diagnostic and Statistical Manual of Mental Disorders (fifth edition) criteria as those children who fail to meet expected developmental milestones in several areas of intellectual functioning at less than five years of age [26]. ^9^ Congenital Anomalies were classified by organ system (metagroup) using the European Registry of Congenital Anomalies and Twins (EUROCAT) Version 2014 [21]. Number of organ systems involved is noted above. ^10^ Examples of technology assistance: tracheostomy tube, ventilation, a feeding tube, a wheelchair or other technology assistance. ^11^ Start date of testing period = Date of first clinical genetics consultation. Median start date for both children with a confirmed genetic diagnosis and those children with no genetic diagnosis was September, 2009. ^12^ Most common genetic diagnoses among those who received a genetic diagnosis (*n* = 147) in the entire CCP. ^13^ CHARGE syndrome: Coloboma of the eye, Heart defects, Atresia of the choanae, Retardation of growth and development, Genital underdevelopment, Ear abnormalities.

**Table 2:** Quantity, Length of Testing Period and Charges of Genetic Testing Among Cohort ^1^.

	All Subjects		Confirmed Genetic Diagnosis		No Genetic Diagnosis		*p* value ^2^
	*n* = 63		*n* = 30		*n* = 33		
Number of Genetic Tests	4	(2.5–7)	3	(2–4)	6	(4–9)	0.002
Length of Testing Period (years)	2.31	(0.33–6.08)	0.35	(0.12–3.04)	4.12	(1.73–8.42)	<0.001
Genetic Testing Costs (C$)	4436	(1869–8726)	2614	(1605–4080)	8496	(4399–12,480)	<0.001

^1^ All values are reflected as median (interquartile range) unless otherwise noted. ^2^ Using the Mann–Whitney U test.
